# EpiTIL: methylation tumor infiltrating lymphocyte (TIL) predictor associates with survival in primary melanoma

**DOI:** 10.1186/s13148-026-02226-6

**Published:** 2026-08-13

**Authors:** Sharon N. Edmiston, Siyao Liu, David L. Corcoran, Honglin Hao, Eloise A. Parrish, David W. Ollila, Paul B. Googe, Pei Fen Kuan, Ronglai Shen, Joel S. Parker, Irene Orlow, Klaus J. Busam, Cecilia Lezcano, Kathleen Conway, Marianne Berwick, Nancy E. Thomas

**Affiliations:** 1 Department of Dermatology, School of Medicine, University of North Carolina at Chapel Hill, Chapel Hill, NC USA; 2 Lineberger Comprehensive Cancer Center, University of North Carolina at Chapel Hill, Chapel Hill, NC USA; 3 Department of Genetics, School of Medicine, University of North Carolina at Chapel Hill, Chapel Hill, NC USA; 4 Department of Surgery, School of Medicine, University of North Carolina at Chapel Hill, Chapel Hill, NC USA; 5 Department of Applied Mathematics and Statistics, State University of New York, Stony Brook, NY USA; 6 Department of Epidemiology and Biostatistics, Memorial Sloan Kettering Cancer Center, New York, NY USA; 7 Department of Pathology and Laboratory Medicine, Memorial Sloan Kettering Cancer Center, New York, NY USA; 8 Department of Epidemiology, School of Public Health, University of North Carolina at Chapel Hill, Chapel Hill, NC USA; 9 Department of Internal Medicine and the UNM Comprehensive Cancer Center, Albuquerque, NM USA

**Keywords:** Tumor infiltrating lymphocytes, Pathology, Skin cancer, Melanoma, DNA methylation, Epigenetics, Immunotherapeutic response prediction

## Abstract

**Background:**

Melanomas are immunogenic with significant variation of tumor infiltrating lymphocytes (TIL). Primary melanomas are routinely scored by pathologists, for TIL grade, as brisk, nonbrisk, and absent, but TIL grade is not utilized in staging. While brisk TIL grade predicts improved melanoma-specific survival, nonbrisk TIL grade often lacks prognostic significance. A high proportion of primary melanomas from stage II and III patients are scored as nonbrisk TIL grade. Our goal is to quantitatively predict T cell estimates in primary melanoma from DNA methylation data using immunofluorescence CD3 + staining as ground truth.

**Results:**

Primary cutaneous melanomas (n = 80), analyzed for multiplex-immunofluorescence (CD3, CD8, S100) and whole-genome DNA methylation, underwent elastic net modeling of the proportion of CD3 + T lymphocytes to build a quantitative prediction model for TILs in primary melanoma called EpiTIL. Melanoma-specific survival (MSS) Kaplan–Meier curves significantly differed by primary melanoma EpiTIL tertiles (*p* = 0.001). Patients with intermediate or highest tertile EpiTIL scores had higher median probability of MSS than those with lowest tertile scores. EpiTIL added significant prognostic value for MSS beyond age, sex, and stage, analyzed using proportional hazard regression modelling (*p* = 0.012). EpiTIL survival prediction was validated in two multi-omic studies, TCGA (n = 352) and InterMEL (n = 399). Among TCGA melanomas, the survival curves significantly differed by EpiTIL tertile scores (*p* = 0.009) and EpiTIL scores added prognostic value to clinical factors (*p* < 0.002). In the InterMEL case–control study of stage II/III melanoma, patients with 5-year MSS without recurrence (controls) had a significantly higher mean EpiTIL scores than those who died of melanoma within 5 years (cases) (*p* = 1.5e − 11). Using logistic regression, EpiTIL scores significantly predicted 5-year MSS without recurrence, even after adjusting for age, sex, stage and TIL grade, with OR = 4.7 (*p* = 2.38e-06) comparing the highest to lowest EpiTIL tertiles. Extending the EpiTIL prediction to the Newell metastatic melanoma cohort, we found immune checkpoint inhibitor responders had a significantly higher mean EpiTIL score than non-responders (*p* = 0.047).

**Conclusion:**

EpiTIL is a DNA methylation-based predictor of T cell proportion that demonstrates potential prognostic value in primary melanoma, particularly for stages II and III. Furthermore, higher EpiTIL scores derived from primary melanomas were positively associated with improved therapeutic responses to immune checkpoint inhibitors in metastatic disease, highlighting its potential to identify patients most likely to benefit from immunotherapy.

**Supplementary Information:**

The online version contains supplementary material available at https://doi.org/10.1186/s13148-026-02226-6.

## Background

Melanomas are immunogenic with significant variation of tumor infiltrating lymphocytes (TIL) [1]. TIL grade, scored by dermatopathologists as brisk, nonbrisk, and absent, is routinely measured in primary tumors but not utilized in staging [2]. While brisk TIL grade predicts improved melanoma-specific survival (MSS), nonbrisk TIL grade often lacks prognostic significance [3, 4]. A high proportion of primary melanomas from stage II and III patients score as nonbrisk TIL grade [5]. Since stage II and III melanoma patients may be considered for immune checkpoint inhibitor (ICI) therapy and CD8 + T cells in the invasive margin predict response to PD-1 checkpoint inhibitors [6, 7], a means to more quantitatively measure T cells than TIL grade would be useful.

Epigenetic regulation of cell-specific differentiation, maturation or functional changes, is responsible for a diverse immune response [8]. DNA methylation, an epigenetic mechanism, is known to regulate gene expression through methylation at CpG sites [9]. DNA methylation at promoter regions regulates T cell activation and differentiation [10, 11], as well as antigen presentation through changes in transcription factor binding [12–15]. Previously, we published that hypermethylation at promoter regions of immune related genes such as HLA markers and immune checkpoint markers was associated with reduced TILs in melanomas and negatively associated with mRNA expression within the CpG Island Methylator Phenotype [16]. These data suggest that DNA methylation is an epigenetic regulator of immune response. Thus, identification of differentially methylated markers associated with immune cells may be harnessed to predict immune cell infiltration.

A methylation TIL signature associated with survival in breast cancer shows limited prognostic ability in advanced melanomas, underscoring the need for a model tailored specifically for melanoma [17]. Given that TIL grade is prognostic in melanoma [3, 18, 19] and immune cells are epigenetically regulated [8], we hypothesized that whole-genome DNA methylation could provide a quantitative prognostic measure of TILs or T cell proportion in primary melanoma. Our goal was to predict T cell estimates in primary melanoma from DNA methylation data using immunofluorescence CD3 + staining as ground truth. In this study, we examined primary melanomas (n = 80) to determine if a model of DNA methylation could predict T cell proportion and stratify risk. The prognostic ability of the resulting model is validated in independent primary melanoma datasets and further evaluated to predict treatment response in an ICI-treated metastatic melanoma cohort.

## Methods

### Patients and tissues

Formalin-fixed, paraffin-embedded (FFPE) primary invasive melanomas were assembled as previously described [20] and included cutaneous primary melanomas from all American Joint Committee on Cancer 2018 (AJCC8) pathological stage patients [2] and a broad spectrum of histologic subtypes. We assembled 80 patients diagnosed between 2001 and 2012 with tumors from the University of North Carolina (UNC) Hospitals’ pathology archives based on original diagnoses abstracted from pathology reports (the UNC-80 cohort). The Institutional Review Board at UNC approved the study and found the research qualified for a waiver of informed consent for research [45 CFR 46.116(d)] and waiver of HIPAA authorization [45 CFR 164.512(i)(2)(ii)].

Multi-omic studies with survival data used for validation included cutaneous primary melanomas from The Cancer Genome Atlas (TCGA) and the InterMEL study. TCGA project [21] has available 352 primary melanomas analyzed for DNA methylation using Infinium Human Methylation 450K arrays (Illumina, Inc.). The InterMEL study, an international case/control study of stage II and III primary melanoma patients, includes 399 patients with whole-genome DNA methylation data (Infinium MethylationEPIC) and complete clinicopathologic data [5, 22].

A metastatic melanoma cohort (n = 77), published by Newell et al., is another multi-omic study that includes DNA methylation data on a subset of patients (n = 43) [23]. The Newell cohort patients were treated with anti-PD-1 monotherapy or combination with anti-CTLA-4 ICI and responses evaluated using Response Evaluation Criteria in Solid Tumors (RECIST 1.1) scores [24]. Patients were considered responders (R) if they scored a complete response (disappearance of tumor and involved lymph nodes) or partial response (≥ 30% of tumor resolved) or a non-responder (NR) if the scores noted stable disease (no significant growth) or progressive disease (≥ 20% growth or new lesions). These data were used to evaluate the EpiTIL model’s ability to predict response to ICI treatment.

### Multiplex immunohistochemistry

All multiplex immunohistochemistry/ immunofluorescence (mIHC/IF) stains (S100_Alexa555; CD3_ Cy5; and CD8_AF488) and quantification of T lymphocytes in melanoma were performed in the UNC Pathology Services Core from whole mount slides as previously described [44]. Using the Definiens Architect XD software to segment tumor tissues, the T cell (CD3 + CD8 + plus CD3 + CD8-) proportion was calculated as the number of T cells divided by the total number of cells within the S100 + -stained melanoma areas.

### DNA methylation analysis

DNA methylation analysis was performed using the genome-wide Infinium HumanMethylation450 array data (450K) (Illumina, Inc.) on the UNC-80 cohort of primary melanomas as described previously [20]. Methylation data is located at Gene Expression Omnibus accession number GSE120878. The 383K CpG probes remaining in the data after preprocessing were used for these analyses. The scripts for building EpiTIL model and the code for generating the figures from this paper are available here: https://github.com/unclbc/EpiTIL_paper.

### Statistical analysis

#### Building elastic net regression model

CD3 + T cell staining image data was used to calculate the proportion of CD3 + T cells infiltrated in the tumor in the UNC dataset. The formula for calculating the CD3 + T cell proportion in the tumor area is the following:

CD3 + T cell proportion = Total number CD3 + T cell count / tumor area cell count

Image analysis is isolated to the melanoma area as defined by Definiens Dx. CD3 + cell counts and total number of cells in the tumor are abstracted from the tumor area.

where:

Total CD3+ T cell count = Number of CD8 − cells co-expressing CD3+ and nuclear markers + Number of CD8+ cells co-expressing CD3+ and nuclear markers

Tumor area cell count = Number of cells expressing nuclear markers

Due to the skewed distribution of CD3 + T cell proportion in the samples, we applied a shifted log 10 transformation on the data using a shift factor 0.01 prior to training the elastic net regression model. For the lambda parameter, glmnet chose the best lambda for each alpha in each model using the cv.glmnet() function based on the mean squared error (mse) measure [25]. To determine the optimal alpha parameter, we used tenfold cross validation to evaluate the model performance using R/glmnet package (v.4.1) and calculated mean squared error between predicted and actual CD3 T cell proportion. We identified the best alpha to be 0.1. The final model was built using the whole dataset and estimated 168 features, or probes, with non-zero coefficients. We identified the top 20 Gene Ontology (GO) [26, 27] terms associated with these final 168 probes using R/topGO(v.2.54) package (http://www.mpi-sb.mpg.de/~alexa). The final model (EpiTIL-168) performance was compared with other published methods, EpiDISH [28, 29] and MethylResolver [30]. In the UNC-80 dataset, we calculated the R squared values within each fold and computed the mean and the standard error of the mean.

#### Survival analysis

The EpiTIL-168 model (168 probes) was applied to the UNC-80. Using the Kaplan–Meier (KM) method [31], we estimated survival curves for both MSS and progression free survival (PFS). The survival curves were displayed by tertiles of predicted EpiTIL scores, and the log rank test was used to examine the differences between the curves. To assess additional variance explained by the predicted EpiTIL score to the prediction of MSS or PFS, we fit a proportional hazard regression model [32] by including clinical variables (age, sex, and stage) and calculated the delta log likelihood ratio test [33] (LLR) using R. The likelihood chi-square statistic was used to assess significance when comparing the deviance of the full model with a reduced model (covariate of interest has been removed).

#### Application of EpiTIL to external melanoma cohorts

For validation, we applied our EpiTIL-168 model to survival prediction in the TCGA dataset. The TCGA data was analyzed as described above for the UNC-80 cohort. We also applied EpiTIL to the InterMEL DNA methylation dataset, limited to the 81 of 168 EpiTIL probes available in this dataset. Further, we tested whether EpiTIL could predict ICI-response in the Newell dataset, limited to the 141 of 168 probes available in this dataset. To evaluate the ability of these more limited probe sets to predict CD3 + T cell proportion, we applied the 81 or 141 probes to our original UNC-80 data and found high correlation with ground truth CD3 + T cell staining data and TIL grade. Box plots were used to visualize and compare distributions within groups for these external cohorts. A Sankey diagram was used to display proportional stratification of TIL grade into EpiTIL tertiles. Wilcoxon tests [34] were performed to examine statistical differences in the means between groups.

TCGA Illumina 450K methylation data can be found at TCGAbiolinks (n = 466) [16] (https://bioconductor.org/packages/release/bioc/html/TCGAbiolinks.html). The InterMEL Illumina Infinium MethylationEPIC data are available through dbGAP (accession number phs003099.v1.p1). The Newell Illumina Infinium MethylationEPIC data [35] can be found at GSE181781.

## Results

### Clinicopathologic characteristics

Primary cutaneous melanomas (n = 80), previously stained for CD3 + T cell expression and screened for global DNA methylation, were used to model T cell estimates in the whole tumor images of the UNC-80 cohort. The UNC-80 cohort patient demographics and clinical and tumor histopathologic characteristics are provided in Table S1. UNC-80 cohort patients were more often male (61.3%), mostly greater than 50 years of age (76%), and represented all AJCC8 pathological stages [Stage I (27.5%); II (40%); III (15%); IV (17.5%)]. The median Breslow thickness of the tumors was 1.6 mm (range, 0.37–17 mm), about a third had ulceration (36.6%) and the majority had a nonbrisk (54%) TIL grade rather than brisk (23%) or absent (23%). After 15 years follow-up, 58.8% of the patients were alive and 41.2% had died of disease.

### Model development to predict T cells in melanoma

**Fig. 1:**
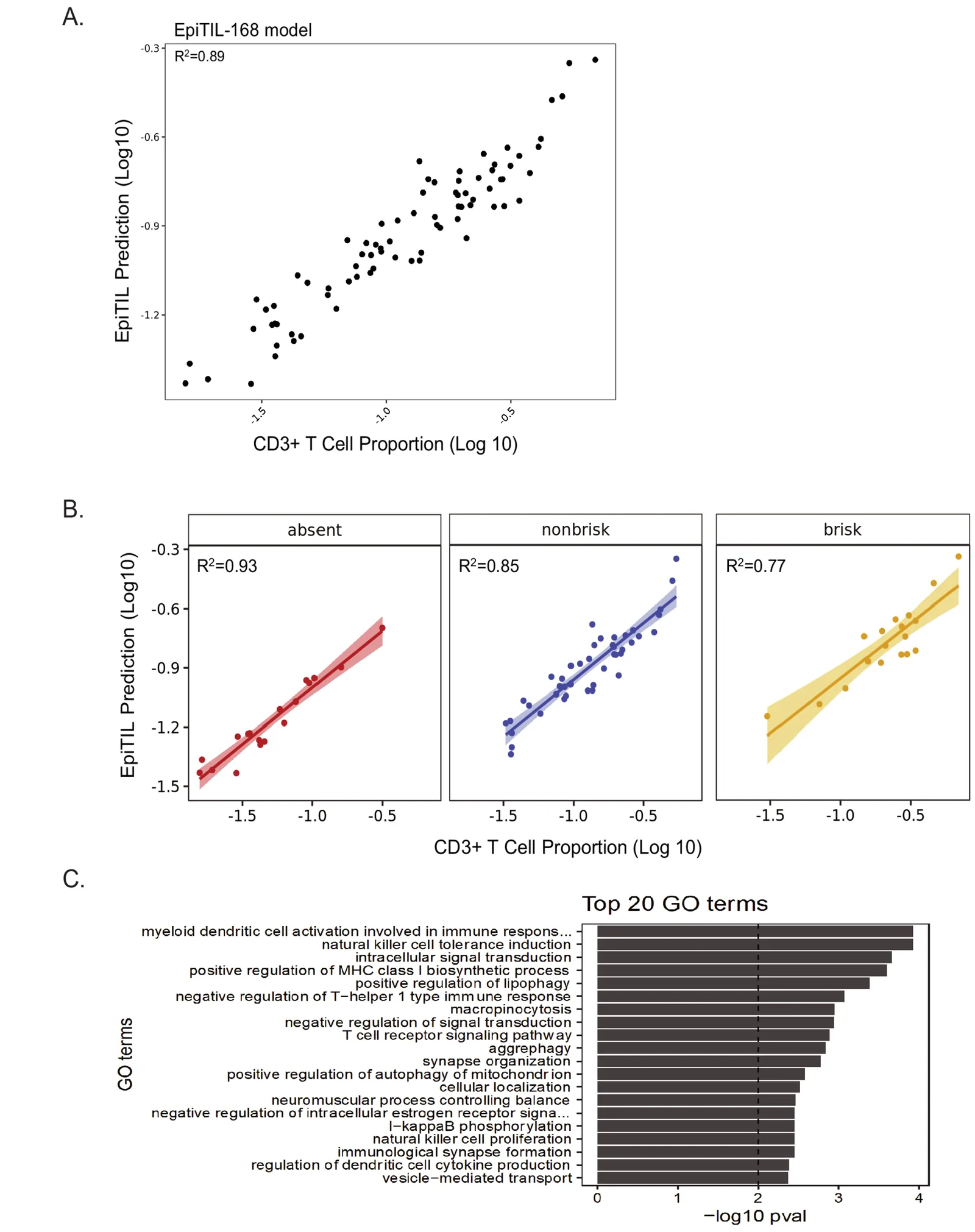
Elastic net modeling proportion of CD3 + T cells in UNC DNA-methylation melanoma samples (n = 80). Graphs showing **A** correlation of EpiTIL-168 prediction (y-axis) with the ground truth CD3 + stained T cell proportion (log10 scale) (x-axis); **B** correlation of EpiTIL-168 prediction (y-axis) with the ground truth CD3 + stained T cell proportion (log10 scale) (x-axis) by TIL grade in UNC dataset; **C** Top 20 Gene Ontology (GO) terms associated with the final elastic net model

Using T cell immunofluorescence (IF) staining data and 450K methylation data, we built a model to predict T cell proportions in melanomas. CD3 + T cell IF image data was used to calculate the proportion of CD3 + T cells infiltrating each tumor in the UNC-80 primary melanoma dataset. Due to the skewed distribution, we applied a shifted log 10 transformation on the data using a shift factor 0.01. We utilized our previously published 450K DNA methylation data (n = 383,693 CpGs) [20] in a tenfold cross validation scheme to experiment and build the prediction model (Figure S1). The model resulting from training on the complete dataset yielded 168 probes with non-zero coefficients. Our epigenetic TIL prediction model (EpiTIL-168) demonstrates high correlation with CD3 + T cell proportion (R^2^ = 0.89) (Fig. 1A) and within each TIL grade [(absent (R^2^ = 0.93; nonbrisk (R^2^ = 0.85); brisk (R^2^ = 0.77)] (Fig. 1B). Gene set enrichment analysis revealed the top 20 GO pathways associated with the 168 CpGs in the EpiTIL-168 model were significantly associated with T cell biology and immune regulatory pathways, such as T cell receptor signaling and myeloid dendritic cell activation involved in immune response pathways (Fig. 1C).

We compared the performance of EpiTIL with other published methods, EpiDISH (*Epi*genetic *D*issection of *I*ntra-*S*ample *H*eterogeneity) [28, 29] and MethylResolver [30]. In the UNC-80 dataset, we calculated the R-squared values within each fold and computed the mean and the standard error of the mean. EpiDISH (R-v2.6.0) allows the identification of cell-type proportions within epigenome-wide association studies (EWAS); specifically, we used the EpiDISH Robust Partial Correlations-RPC specific for the T cell fraction of the whole blood analysis. EpiTIL quantified T cell proportions and displays high correlation with EpiDISH (R^2^ = 0.60) and low correlation with MethylResolver (R^2^ = 0.17) in predicting the T cell proportions in primary melanomas (Fig. S2).

### EpiTIL associates with survival in UNC-80

**Fig. 2:**
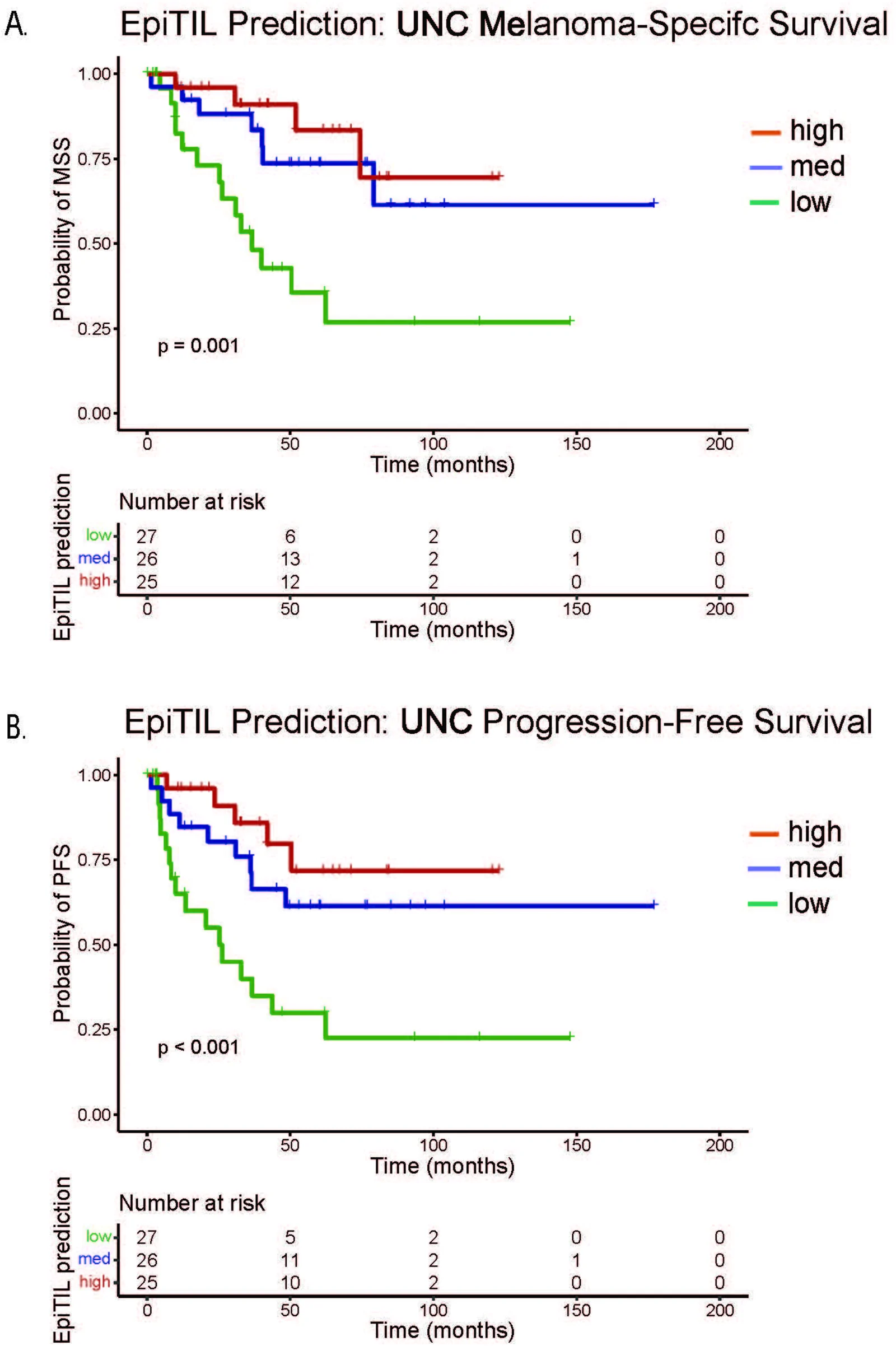
Kaplan–Meier (KM) survival probabilities by EpiTIL tertiles in the UNC-80 dataset. **A** KM curves of MSS using tertiles defined by EpiTIL-168 prediction. **B** KM curves of PFS using tertiles defined by EpiTIL-168 prediction

In the UNC-80 cohort, we produced KM survival curves for MSS using EpiTIL-168 scores. EpiTIL-168 scores were displayed as tertiles in KM curves and compared using a log rank test (LRT) (Fig. 2). MSS differed significantly across EpiTIL tertiles (LRT, *p* = 0.001) (Fig. 2A) in patients with the intermediate and highest EpiTIL-168 tertiles demonstrating a higher probability of MSS compared with those in the lowest tertile scores. To determine if the EpiTIL-168 scores provided prognostic MSS information beyond standard clinical factors, we compared the full model (sex, age, stage, and EpiTIL-168) with a reduced model (EpiTIL, covariate of interest removed). We found that EpiTIL-168 scores significantly improved MSS prediction compared with the clinical model (LLR, *p* = 0.01). EpiTIL positive association with improved MSS prediction remains significant even when TIL grade is included in the models (LLR, *p* = 0.02).

Increasing EpiTIL-168 scores were also associated with differences in KM curves for progression-free survival (PFS) (LRT, *p* < 0.001) (Fig. 2B). Patients with tumors having the highest or intermediate EpiTIL-168 tertile scores had higher probability of PFS compared to those with the lowest tertile scores.

### Comparison of EpiTIL with EpiDISH in UNC-80

Given that EpiTIL-168T cell prediction correlated with EpiDISH proportions of T cells, we conducted a survival analysis using EpiDISH in the UNC-80 cohort. We examined survival using the KM method with the EpiDISH model and found no significant differences in survival curves associated with MSS (LRT, *p* = 0.510) (Fig. S3A) or PFS (LRT, *p* = 0.400) (Fig. S3B). Survival prediction was not improved by EpiDISH compared to the model including clinical variables, age, sex, and stage (MSS, *p* = 0.394; PFS, *p* = 0.233) (Fig. S3C, D, respectively).

### EpiTIL validation in TCGA

Using the external TCGA melanoma dataset, we found the survival curves significantly differed by EpiTIL-168 tertile scores (LRT, *p* = 0.009). Patients with the highest tertile EpiTIL-168 scores show higher probability of MSS than those with intermediate or lowest tertile scores (Fig. S4). To assess additional variance explained by the EpiTIL-168 score for the prediction of MSS in TCGA, we performed analysis including stage, age and sex. EpiTIL-168 tertile scores improved our ability to estimate MSS compared to a clinical model using age, sex and stage, alone (LLR, *p* = 0.002).

### EpiTIL validation in InterMEL

We further validated our EpiTIL-168 model in an independent DNA methylation dataset from the international InterMEL case–control study, which includes primary melanomas from stage II/III patients who were not treated with modern adjuvant therapies [5]. In InterMEL, cases are patients who died from melanoma within 5 years, and controls are those who lived for at least 5 years after diagnosis without recurrence. Of the EpiTIL-168 CpGs, 81 were available in the InterMEL dataset. To confirm that these 81 CpGs predicted T cell proportion, we applied the 81 CpGs back onto the UNC-80 cohort and found these 81 CpGs were highly correlated with T cell proportion (R^2^ = 0.87) (Fig. S5A) and within each TIL grade [absent (R^2^ = 0.95); nonbrisk (R^2^ = 0.80); brisk (R^2^ = 0.74)] (Fig. S5B). Thus, based on the preserved performance, we applied the EpiTIL-81 prediction model to the InterMEL dataset to predict T cell proportion and evaluate an association with survival by case–control status. Since TMB may be associated with immune infiltrate [36, 37] and reported to predict response to ICI [38], we examined EpiTIL with TMB in the InterMEL study [39]. EpiTIL did not correlate with TMB (R^2^ = 0.049, *p* = 0.38). Further, we found no association of EpiTIL score with BRAF, NF1, NRAS, or TripleWT status (BRAF *p* = 0.62; NF1 *p* = 0.39; NRAS *p* = 0.47; TripleWT *p* = 0.20) using Wilcoxon sign-ranked test to compare melanoma with and without individual gene mutations.

**Fig. 3:**
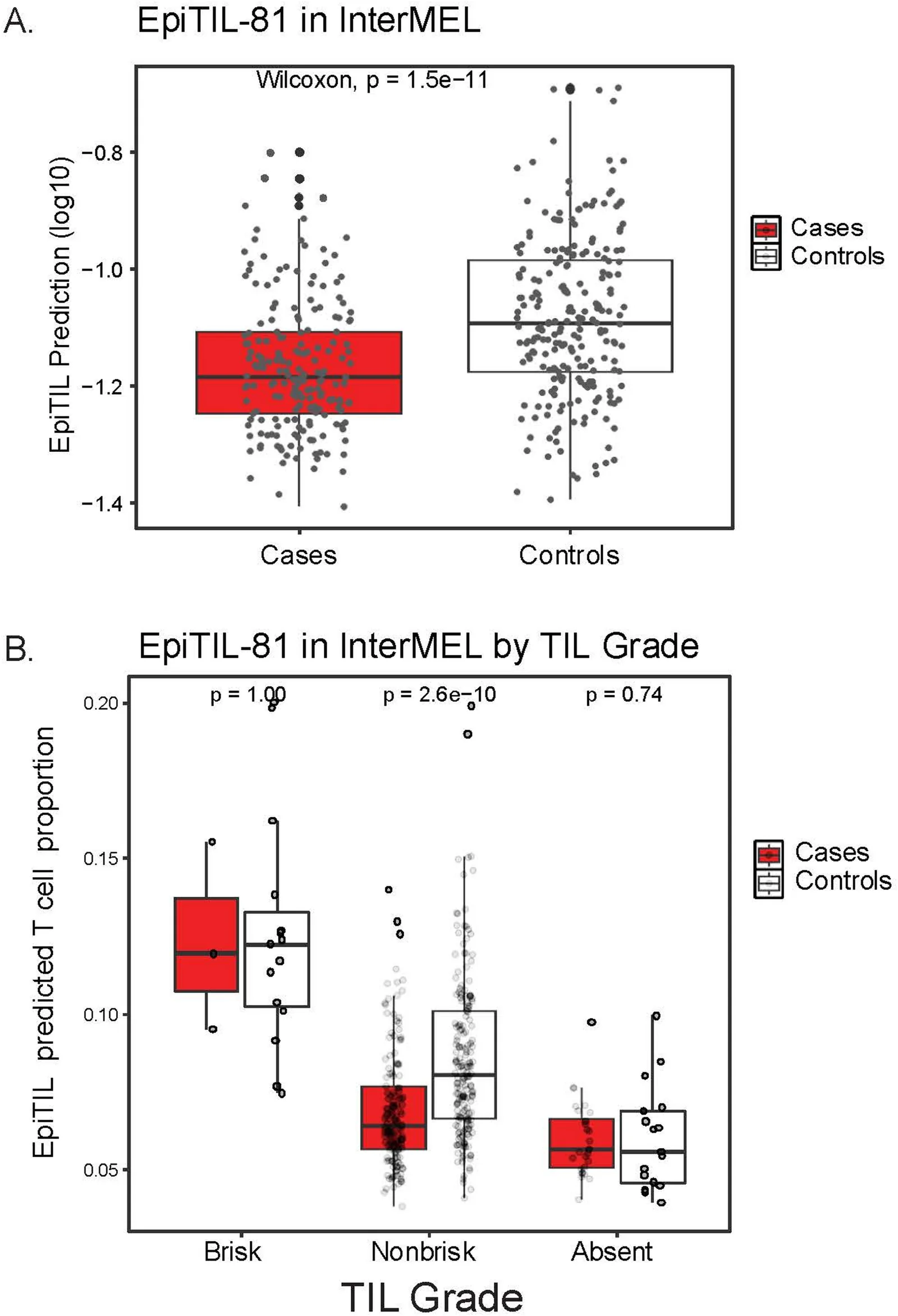
Validation of EpiTIL prediction in InterMEL dataset. **A** Box plot of EpiTIL-81 prediction in InterMEL dataset by case (died < 5yrs. after diagnosis) and control (alive > 5yrs. after diagnosis). **B** Box plot of EpiTIL-81 prediction in InterMEL dataset by case (died < 5yrs. after diagnosis) and control (alive > 5yrs. after diagnosis) within TIL grade

InterMEL controls had a higher mean EpiTIL-81 score than cases (Wilcoxon *p* = 1.5e-11) (Fig. 3A). Within the nonbrisk TIL grade group, the EpiTIL-81 scores showed significant differences between case–control status, with controls having higher a mean EpiTIL-81 score than cases (Wilcoxon *p* = 2.6e-10) (Fig. 3B). As shown in box plots of the EpiTIL tertiles versus TIL grades, fewer patient tumors fell within the intermediate EpiTIL-81 tertile than within the nonbrisk TIL grade category (Fig. S6), potentially related to improved quantitation of TILs. A Sankey diagram displays the proportional stratification of TIL grade by EpiTIL tertile. Melanomas with nonbrisk TIL grade were stratified into high, intermediate or low EpiTIL tertiles illustrating that the TIL grade assigned by the pathologist visually has a large variation in the nonbrisk category. Also, melanomas scored as absent TIL grade display a few samples with high EpiTIL scores and several with intermediate EpiTIL scores, perhaps because the immune infiltrate is difficult to visualize with layering of tumor cells (Fig. S7).

To examine survival in the InterMEL study, odds ratios were calculated comparing cases to controls using EpiTIL-81 scores for 5-year MSS (Table 1). Patients in the highest or intermediate EpiTIL-81 tertile showed an increased odds of 5-year survival without recurrence compared to those in the lowest tertile (OR, 6.54 and 3.56 for the highest and intermediate tertiles, respectively, both *p* < 0.001) adjusted for age and sex. Since EpiTIL scores decrease with increasing stage (*p* = 5.1e-06) and T stage (*p* = 2.5e-09), we decided to include stage in the model. The association remained significant after adjusting for age, sex and stage (ORs, 5.07 and 3.27 for the highest and intermediate tertiles, respectively, both *p* < 0.001) and after adjusting for age, sex, stage and TIL grade (ORs, 4.72 and 3.21 for the highest and intermediate tertiles, respectively, both *p* < 0.001). In comparison for InterMEL, we calculated odds ratios for 5-year melanoma-specific survival by TIL grade. Patients have better odds of 5-year survival with brisk (OR = 7.6, *p* = 0.005) and nonbrisk TIL grades (OR = 1.94, *p* = 0.05). However, these odds were attenuated and do not reach statistical significance when adjusting for stage in the model (brisk TIL OR = 4.41, *p* = 0.06; nonbrisk TIL OR = 1.63, *p* = 0.20). (Table S3).

**Table 1:** InterMEL Study: Odds ratios for 5 years melanoma-specific survival

Groups compared	Odds Ratio	95% CI	p value
EpiTIL subclass (age + sex)^1^			
EpiTIL (low)	ref		
EpiTIL (IM)	3.56	1.52–4.22	3.65E-04
EpiTIL (high)	6.54	3.55–10.40	5.99E-11
EpiTIL subclass (age + sex + stage)^2^			
EpiTIL (low)	ref		
EpiTIL (IM)	3.27	1.49–4.78	1.07E-03
EpiTIL (high)	5.07	2.61–8.68	4.08E-07
EpiTIL subclass (age + sex + stage + TIL grade)^3^			
EpiTIL (low)	ref		
EpiTIL (IM)	3.21	1.46–4.76	1.35E-03
EpiTIL (high)	4.72	2.45–8.63	2.38E-06

CI, Confidence Interval; IM, Intermediate; TIL, Tumor Infiltrating Lymphocytes

^1^Base model adjusted for age, sex; ^2^Base model + AJCC stage; ^3^Base model + AJCC stage + TIL grade (absent, nonbrisk, brisk). Categorical descriptions: American Joint Committee on Cancer (AJCC); AJCC Category (Cat.) (Gershenwald et al., CA Cancer J Clin. 2017) Cat. 1: IIA + IIIA, Cat. 2: IIB + IIC + IIIB, Cat. 3: IIIC, Cat. 4: IIID; and TIL grade (absent, nonbrisk, brisk) (Clark et al., JNCI, 1981; Busam et al., Am J Clin Pathol 2001)

### EpiTIL associates with response to ICI therapy

**Fig. 4:**
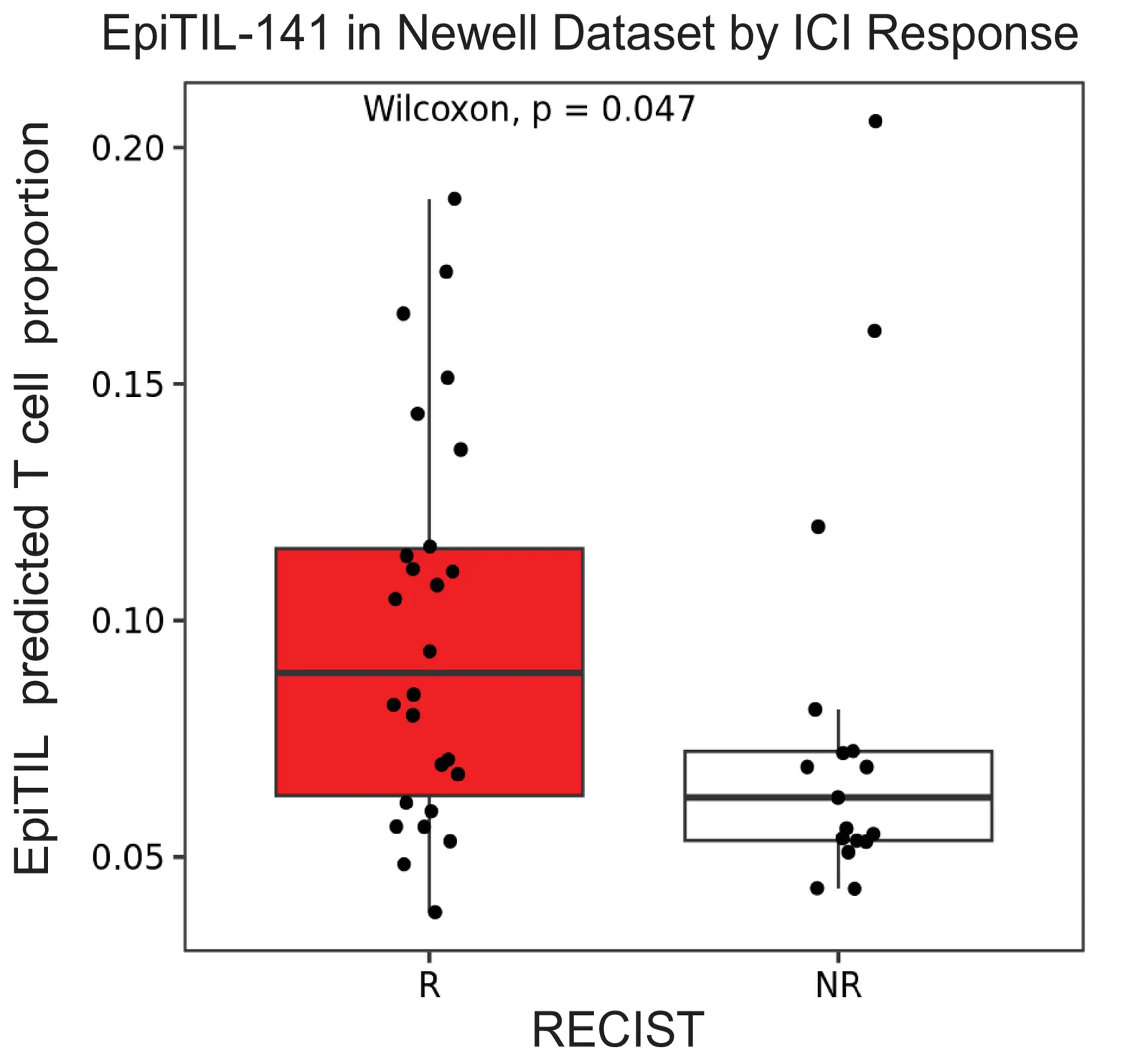
EpiTIL prediction associates with ICI therapy response in Newell dataset. Box plot of EpiTIL-141 prediction by RECIST score categories, responder (R) (complete and partial responders) compared to non-responder (NR) (stable and progressive disease) within the Newell metastatic melanoma samples

To investigate whether the EpiTIL-168 model would be a useful tool for determining response to ICI therapy, we examined the metastatic melanoma dataset published by Newell et al. [35]. The Newell cohort patients (n = 77) were treated with anti-PD-1 alone or dual anti-PD-1/anti-CTLA-4 ICIs with 43 samples with DNA methylation data available. Due to the limited study size (n = 43), we examined responders (R) versus non-responders (NR), where responders were patients with complete response and partial response while non-responders were patients with stable disease or progression. Of the EpiTIL-168 CpGs, 141 were available in the dataset. We applied the 141 CpGs onto the UNC-80 cohort and found good correlation with T cell proportion (R^2^ = 0.89) (Fig. S5C) and within each TIL grade [absent (R^2^ = 0.93); nonbrisk (R^2^ = 0.84); brisk (R^2^ = 0.76)] (Fig. S5D). We applied the EpiTIL-141 model to a subset of the Newell dataset (n = 43) with DNA methylation data to predict T cell proportion. EpiTIL-141 scores were associated with RECIST 1.1 response data from patients treated with ICI. Patients who responded to ICI had a significantly higher mean EpiTIL-141 score than non-responders (Wilcoxon, *p* = 0.047) (Fig. 4). To examine response stratified by EpiTIL, we compared EpiTIL tertiles for response and plotted box plots showing ICI responders (R; red) and non-responders (NR; white) plotted by EpiTIL tertiles (high, intermediate, low) (Supplementary Fig. S8). The positive trend of increasing EpiTIL association with ICI response remains for the low and intermediate EpiTIL groups but the significance was attenuated (Fig. S8A). This is probably due to small sample size as some categories contain less than 10 samples. We hope to extend EpiTIL to a larger dataset in future studies. We also examined ICI response by anti-PD1 monotherapy and combination anti-PD1/anti-CTLA-4 ICI therapy and found significant response in patients treated with combination therapy (*p* = 0.047) compared to monotherapy (Fig. S8).

## Discussion

We developed EpiTIL, a methylation TIL predictor trained on the proportion of CD3 + T cells in primary melanoma. EpiTIL demonstrated consistent prognostic value in two validation cohorts. Specifically, EpiTIL-168 scores associated with MSS in TCGA. In the international InterMEL study, patients with the highest and intermediate EpiTIL-81 tertiles had a 4.7- and 3.2-fold, respectively, better odds of 5-year survival from melanoma without recurrence adjusting for age, sex, stage and TIL grade. Together, these findings indicate that a DNA methylation-based, immune-related signature, specifically trained on primary melanomas, shows promise as a prognostic indicator for melanoma outcomes.

Our data highlights the value of genome-wide analysis for identifying CpGs associated with T cell proportions in primary melanomas. Both EpiTIL and EpiDISH show good correlation with IF-stained CD3 + T cells. However, only EpiTIL, derived from genome-wide methylation data in primary melanoma, proved to be prognostic for primary melanoma, whereas EpiDISH, relying on cell-type specific reference data generated from blood, lacked prognostic capability. Leveraging the complete array of CpGs may have aided in developing a signature that reflects epigenomic regulatory events influencing both tumor biology and T cell growth, attraction, and tumor infiltration or exclusion, important in identifying the proportion of T cell infiltration into the tumor and survival prediction. EpiTIL contains features from multiple biological pathways and immune pathways other than T cell density. The top Gene Ontology biological pathways identified with EpiTIL markers include myeloid dendritic cell activation involved in immune response, natural killer cell tolerance induction, intracellular signal transduction, positive regulation of MHC I biosynthetic processes, and positive regulation of lipophagy. Lipophagy refers to the process of breaking down lipids for cellular metabolism indicating that EpiTIL contains features associated with pathways other than immune pathways.

EpiTIL tertile scores were also associated with response to ICI therapy in the Newell et al. metastatic cohort [23]. Our results support previously published data showing an increased proportion of CD8 + T cells are associated with improved response to ICI in metastatic melanoma [23]. Ressler et al. [40] examined tumors from metastatic melanoma patients and found DNA methylation associated with ICI response, with responders showing more hypomethylation compared to nonresponders. Further, a study by Klein et al. [41], found that ICI response was linked to increased TIL clusters in *BRAFV600* mutant metastatic melanoma compared to *NRAS* mutant or wildtype tumors.

To our knowledge, this is the first study that links a T cell predictive model developed in primary melanoma to an ICI response in metastatic melanoma. Previous studies performed in primary melanoma found no association of TIL grade with ICI response [42, 43]. For instance, a primary melanoma study comparing traditional TIL grade with ICI response found no association with ICI response although brisk TIL grade associated with recurrence-free survival [42]. Similarly, van Duin et al. reported that TILs in primary melanoma were not predictive of ICI response [43]. In contrast, our primary melanoma EpiTIL scores were associated with ICI response in metastatic disease. We found EpiTIL associated with multiple immune pathways suggesting that it may capture broader, more comprehensive features of the anti-tumor immune response than TIL grade or TIL density alone. Thus, EpiTIL warrants further study as a biomarker in primary melanoma to predict ICI response, particularly given the FDA approval of ICI therapy for stage II B/C melanomas in 2021 [6].

Our study is notable for identifying a DNA methylation signature that predicts T cell proportions and indicated prognostic value in stage II/III primary melanoma. The EpiTIL signature reflects epigenetic regulation across a diverse set of immune-related pathways that may be a more comprehensive measure of immune response than TIL grade, while offering a more precise measure of T cell density. There are some limitations in our study. First, incomplete overlap of CpG sites across all datasets results in a reduced model in some external cohorts. Second, the Newell cohort used to examine ICI response is relatively small (methylation data available for 43/77 patients) limiting power to characterize therapeutic response in greater detail. While TIL grade is useful in some melanoma cohorts with a larger percentage of brisk TIL grade, our results suggest that stage II and III melanomas with nonbrisk TIL grade may benefit most from EpiTIL scoring. EpiTIL may need further evaluation in more cohorts to evaluate the full scope of application.

## Conclusion

EpiTIL is a DNA methylation predictor of T cell proportion in primary melanoma that demonstrates potential prognostic value for melanoma outcomes. Importantly, EpiTIL scores, derived from primary melanoma, are also associated with ICI therapeutic response in metastatic disease. These findings suggest that EpiTIL may help to identify patients most likely to benefit from immunotherapy and support its further evaluation as a marker to inform risk stratification and treatment decision-making in melanoma.

## Supplementary Information

Supplementary file 1.

## Data Availability

UNC-80 methylation data is located at Gene Expression Omnibus accession number GSE120878. TCGA Illumina 450 K methylation data can be found at TCGAbiolinks (n = 466) [16] (https://bioconductor.org/packages/release/bioc/html/TCGAbiolinks.html). The InterMEL Illumina Infinium MethylationEPIC data are available through dbGAP (accession number phs003099.v1.p1). The Newell Illumina Infinium MethylationEPIC data [35] can be found at GSE181781. The scripts for building EpiTIL model and the code for generating the figures from this paper are available here: https://github.com/unclbc/EpiTIL_paper.

## Abbreviations

TIL: Tumor infiltrating lymphocyte
MSS: Melanoma-specific survival
PFS: Progression-free survival
mIHC/IF: Multiplex immunohistochemistry/immunofluorescence
ICI: Immune checkpoint inhibitor
