# Supplementary material for: EpiTIL: methylation tumor infiltrating lymphocyte (TIL) predictor associates with survival in primary melanoma
Source: Clin Epigenetics. 2026 Aug 13;18:209. doi: 10.1186/s13148-026-02226-6 (PMC13750054; doi:10.1186/s13148-026-02226-6)
Supplement: Supplementary file 1 [file 13148_2026_2226_MOESM1_ESM.pdf]

EpiTIL: Methylation tumor infiltrating lymphocyte (TIL) predictor associates with survival in primary melanoma

Supplementary Figures and Tables

Sharon N. Edmiston[1,2,3], Siyao Liu[3], David L. Corcoran [2,3], Honglin Hao[1], Eloise A. Parrish[1,2], David W. Ollila[2,5], Paul B. Googe[1,2], Pei Fen Kuan[6], Ronglai Shen[7], Joel S. Parker[2,3], Irene Orlow[7], Klaus Busam[7], Cecilia Lezcano[7], Kathleen Conway[1,2,8] , Marianne Berwick[9], Nancy E. Thomas[1,2]

Figure Legends: Supplement

Figure S1: T Cell Prediction Model. Panel illustrates steps in the process to build a model to predict T cell proportion in melanoma. (A) T Cell IF Data; Representation of multiplex immunofluorescence staining for melanoma (S100+, cyan) and T cells (CD3+, red; CD8+, green). (B) DNA Methylation Data; Heatmap of differentially methylated CpGs associated with melanoma TIL grade. (C) Schematic showing the steps in data processing in the UNC-80 cohort.

Figure S2: Correlation with published T cell methylation models: Box plots showing the correlation of EpiTIL prediction compared with EpiDISH and MethylResolver.

Figure S3: Survival in UNC Cohort using EpiDISH: Kaplan-Meier survival curves showing (A) melanoma-specific survival (DSS) and (B) progression-free survival (PFS) in the UNC-80 melanoma cohort by EpiDISH scores. Model comparisons of EpiDISH with clinical variable model (age, sex, and stage) using log likelihood ratio test for (C) melanoma-specific survival and (D) progression-free survival.

Figure S4: Survival in TCGA Validation Cohort: Kaplan-Meier survival curves showing melanoma-specific survival in the TCGA melanoma cohort by EpiTIL-168 scores.

Figure S5: T Cell Prediction using Reduced EpiTIL models applied to the UNC-80 cohort. (A) EpiTIL-81 prediction correlation with T cell percentage. (B) EpiTIL-81 correlated with TIL grade. (C) EpiTIL-141 predicted scores correlated with T cell percentage. (D) EpiTIL-141 correlation with TIL grade.

Figure S6: Proportion of T cells in InterMEL in Cases and Controls. (A) Bar plots showing the proportion of T cells measured by EpiTIL tertiles in cases and controls. (B) Bar plots comparing TIL grade in cases and controls.

Figure S7: Sankey diagram showing the proportional stratification of TIL grade into EpiTIL tertiles. TIL grade [brisk (green), nonbrisk (blue), absent (red)] are on the right and EpiTIL tertiles (high, intermediate, low) are on the left.

Figure S8: ICI Response in Metastatic Melanoma by EpiTIL scores. (A) Box plots showing ICI responders (R; red) and non-responders (NR; white) plotted by EpiTIL tertiles (high, intermediate, low); p values are listed above each tertile. (B) Box plots showing ICI response by combination therapy (Ipi + PD1) or mono therapy (PD1) for complete response (CR; dark red), partial response (PR; pink), stable disease (SD; dark grey), and progressive disease (PD; light grey) plotted by EpiTIL scores.`

Figure S1

Build a model to predict T cells in melanoma

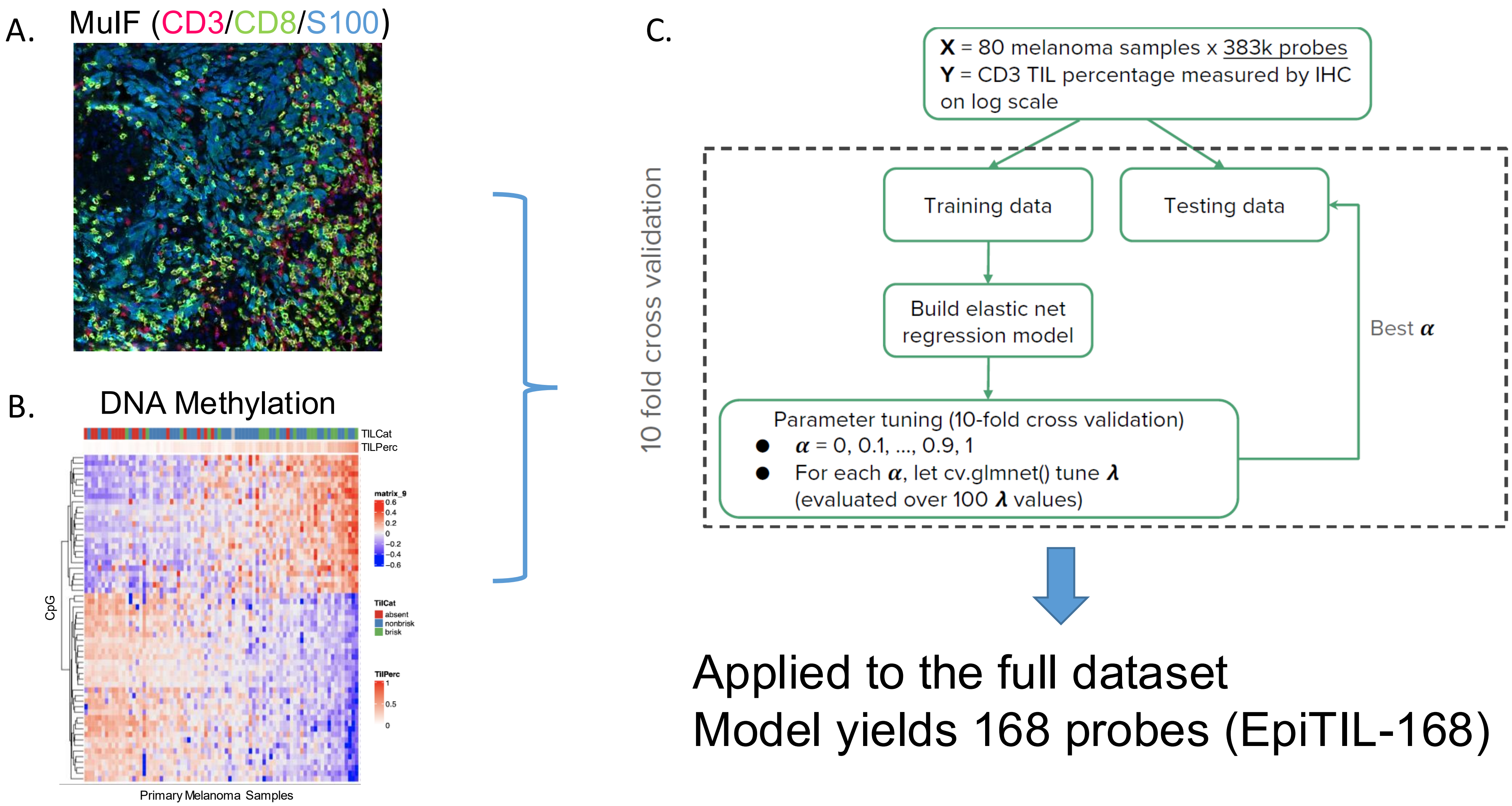

Figure S2

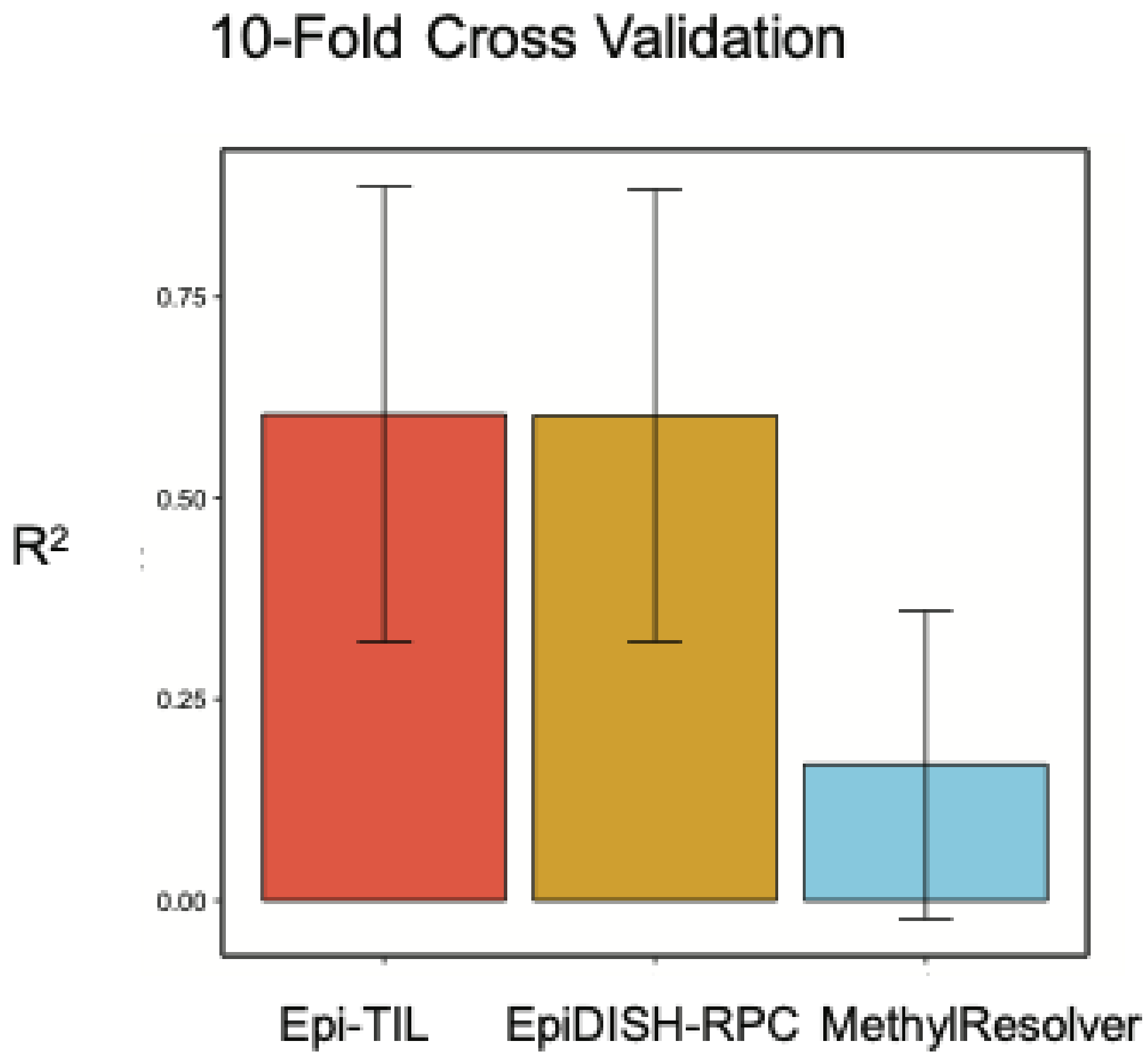
10-Fold Cross Validation
R2
0.75
0.50
0.25
0.00
Epi-TIL
EpiDISH-RPC
MethylResolver

Figure S3

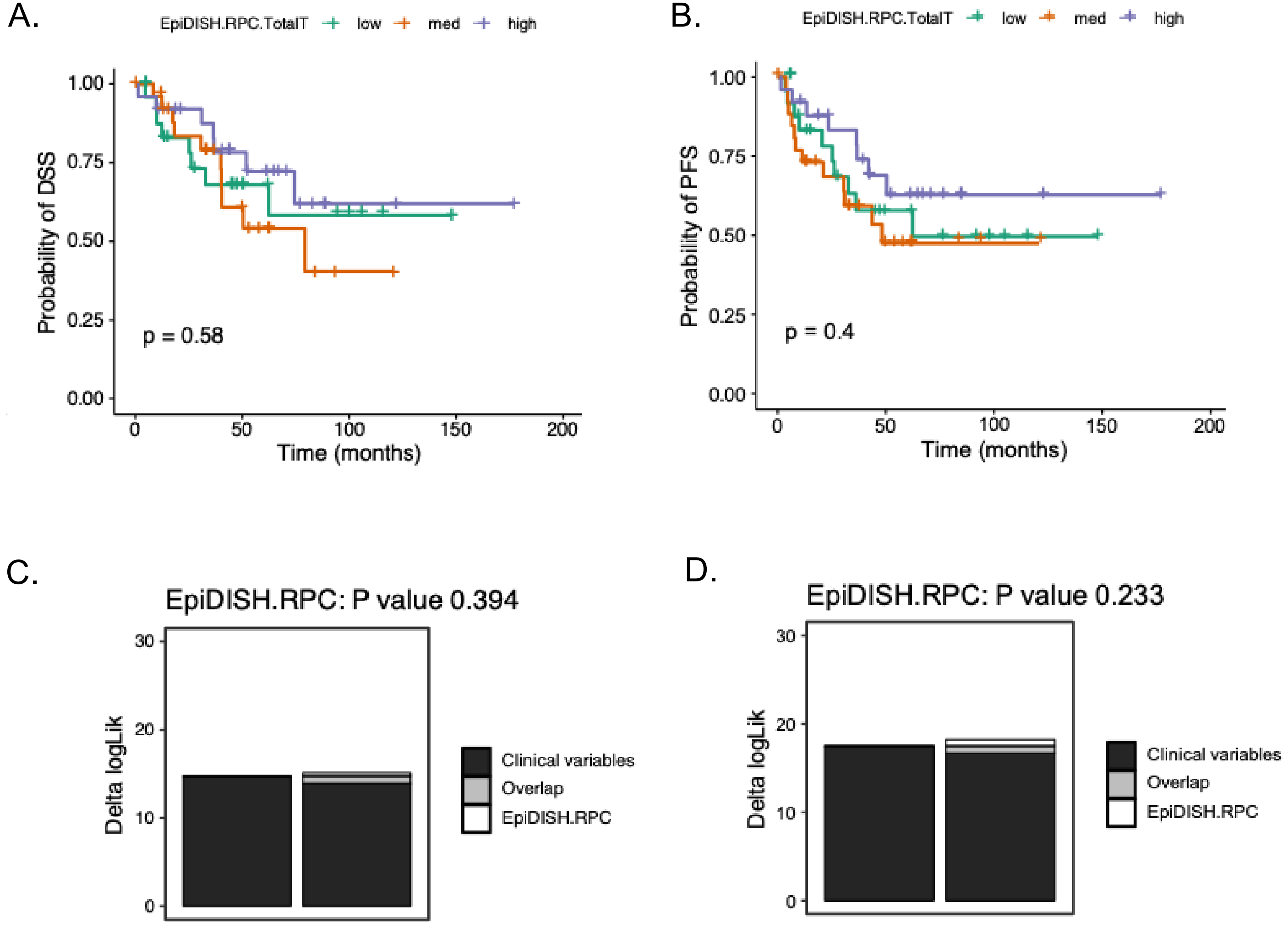
A.
EpiDISH.RPC.TotalT low med high
Probability of DSS
1.00
0.75
0.50
0.25
0.00
p = 0.58
0
50
100
150
200
Time (months)
B.
EpiDISH.RPC.TotalT low med high
Probability of PFS
1.00
0.75
0.50
0.25
0.00
p = 0.4
0
50
100
150
200
Time (months)
C.
EpiDISH.RPC: P value 0.394
Delta logLik
30
20
10
0
Clinical variables
Overlap
EpiDISH.RPC
D.
EpiDISH.RPC: P value 0.233
Delta logLik
30
20
10
0
Clinical variables
Overlap
EpiDISH.RPC

Figure S4

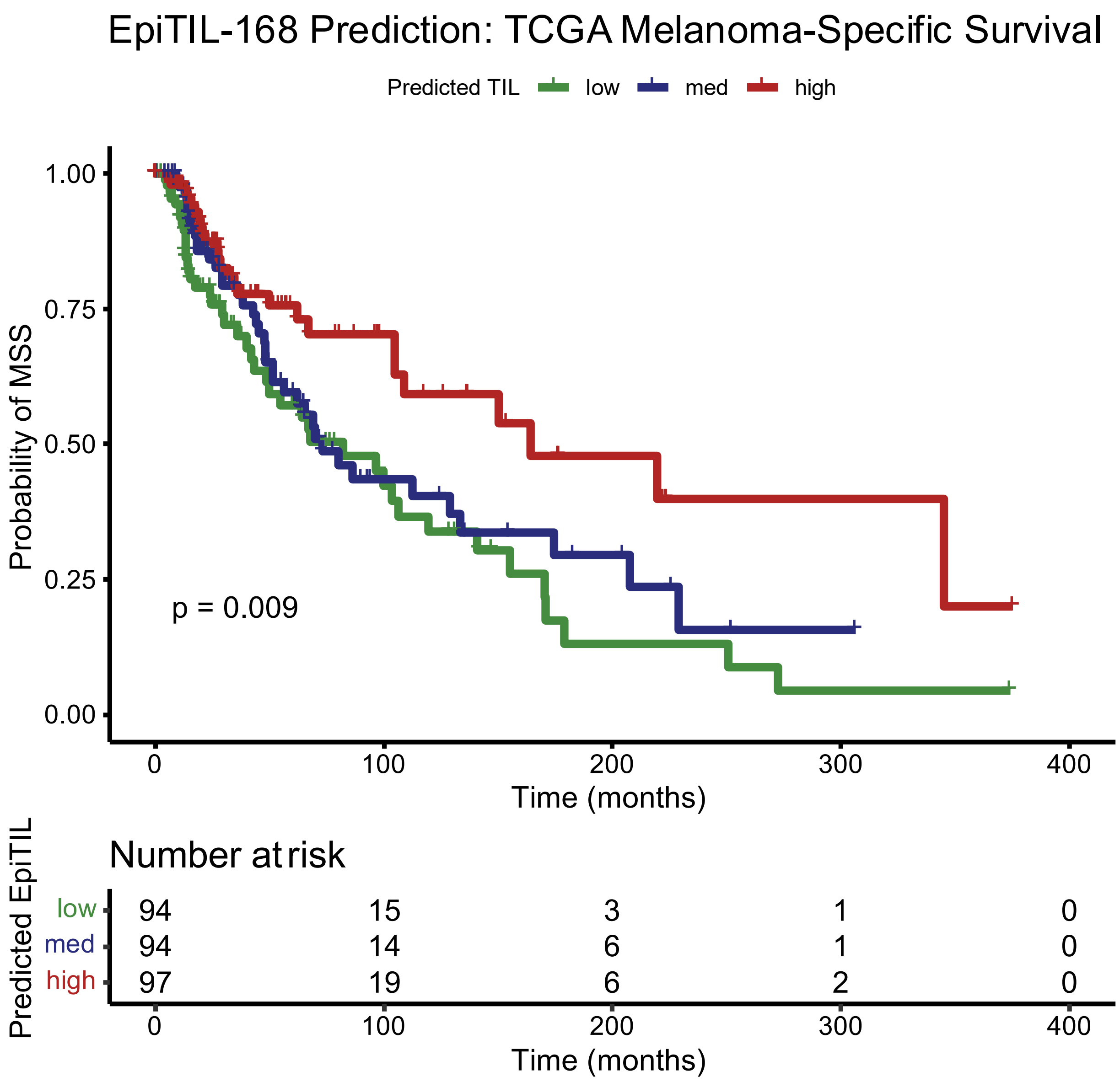

EpiTIL-168 Prediction: TCGA Melanoma-Specific Survival
Predicted TIL
low
med
high
Probability of MSS
1.00
0.75
0.50
0.25
0.00
p = 0.009
0
100
200
300
400
Time (months)
Number at risk
Predicted EpiTIL
low
med
high
94
15
3
1
0
94
14
6
1
0
97
19
6
2
0
0
100
200
300
400
Time (months)

Figure S5

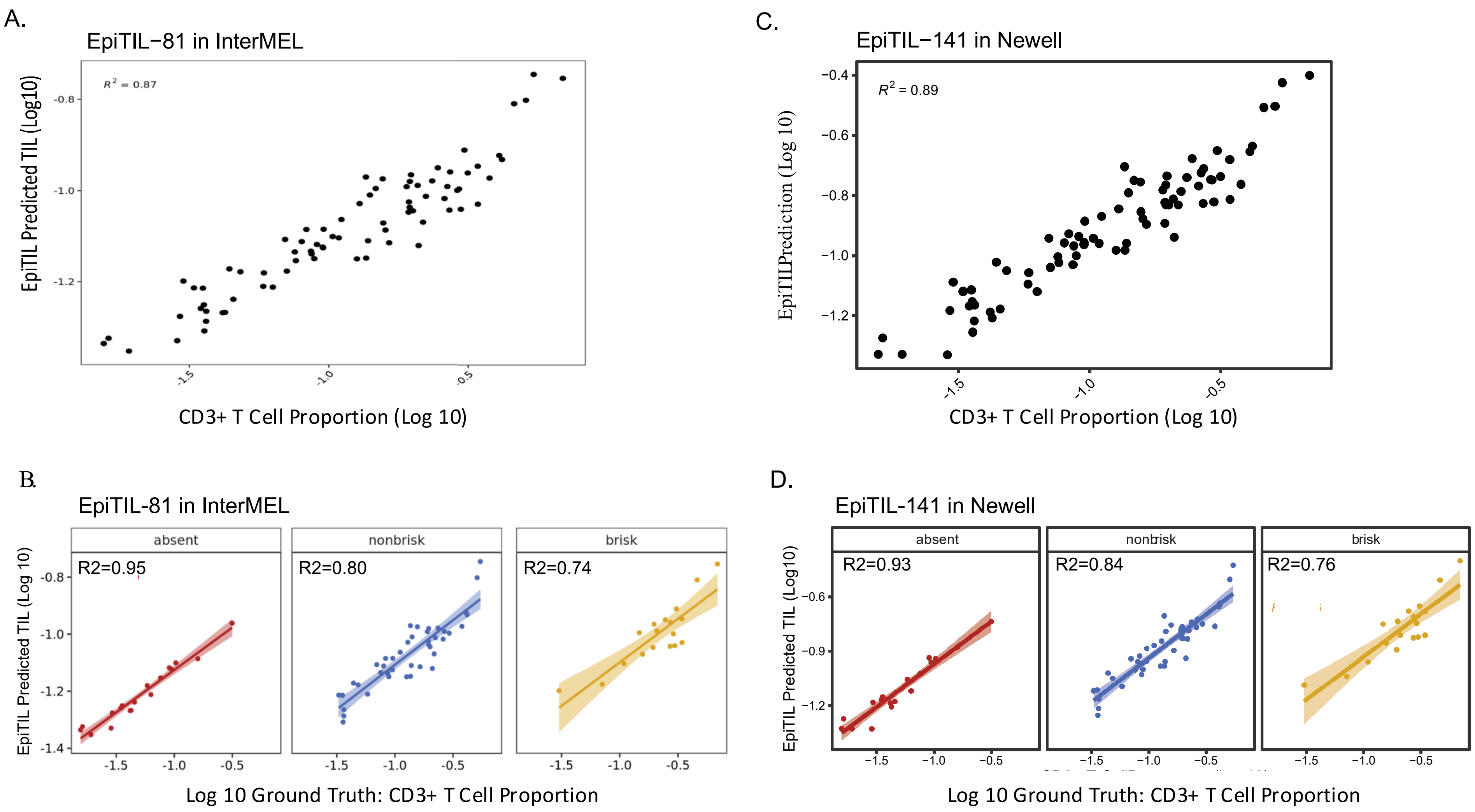
A.
EpiTIL−81 in InterMEL
R^2 = 0.87
EpiTIL Predicted TIL (Log10)
CD3+ T Cell Proportion (Log 10)
C.
EpiTIL−141 in Newell
R^2 = 0.89
EpiTILPrediction (Log 10)
CD3+ T Cell Proportion (Log 10)
B.
EpiTIL-81 in InterMEL
absent
nonbrisk
brisk
R2=0.95
R2=0.80
R2=0.74
EpiTIL Predicted TIL (Log 10)
Log 10 Ground Truth: CD3+ T Cell Proportion
D.
EpiTIL-141 in Newell
absent
nonbrisk
brisk
R2=0.93
R2=0.84
R2=0.76
EpiTIL Predicted TIL (Log10)
Log 10 Ground Truth: CD3+ T Cell Proportion

Figure S6

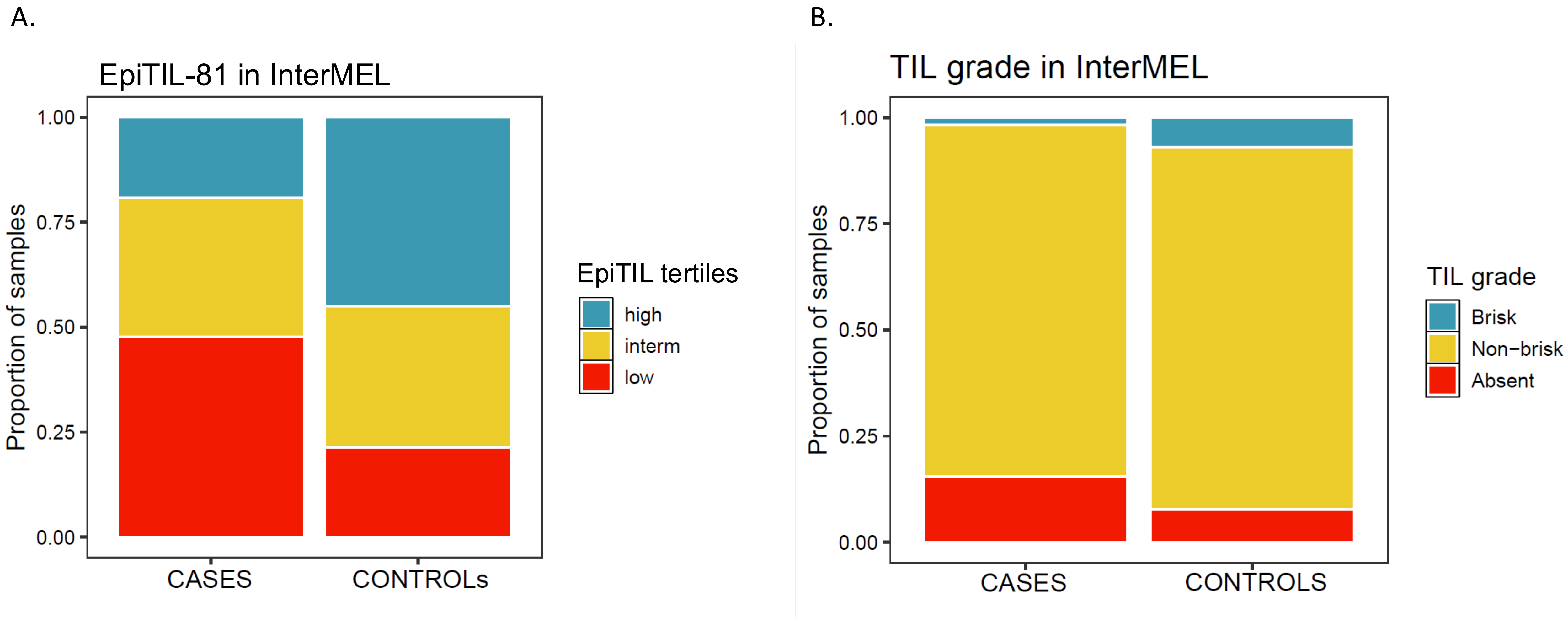
A.
EpiTIL-81 in InterMEL
Proportion of samples
1.00
0.75
0.50
0.25
0.00
CASES
CONTROLs
EpiTIL tertiles
high
interm
low
B.
TIL grade in InterMEL
Proportion of samples
1.00
0.75
0.50
0.25
0.00
CASES
CONTROLS
TIL grade
Brisk
Non−brisk
Absent

Figure S7

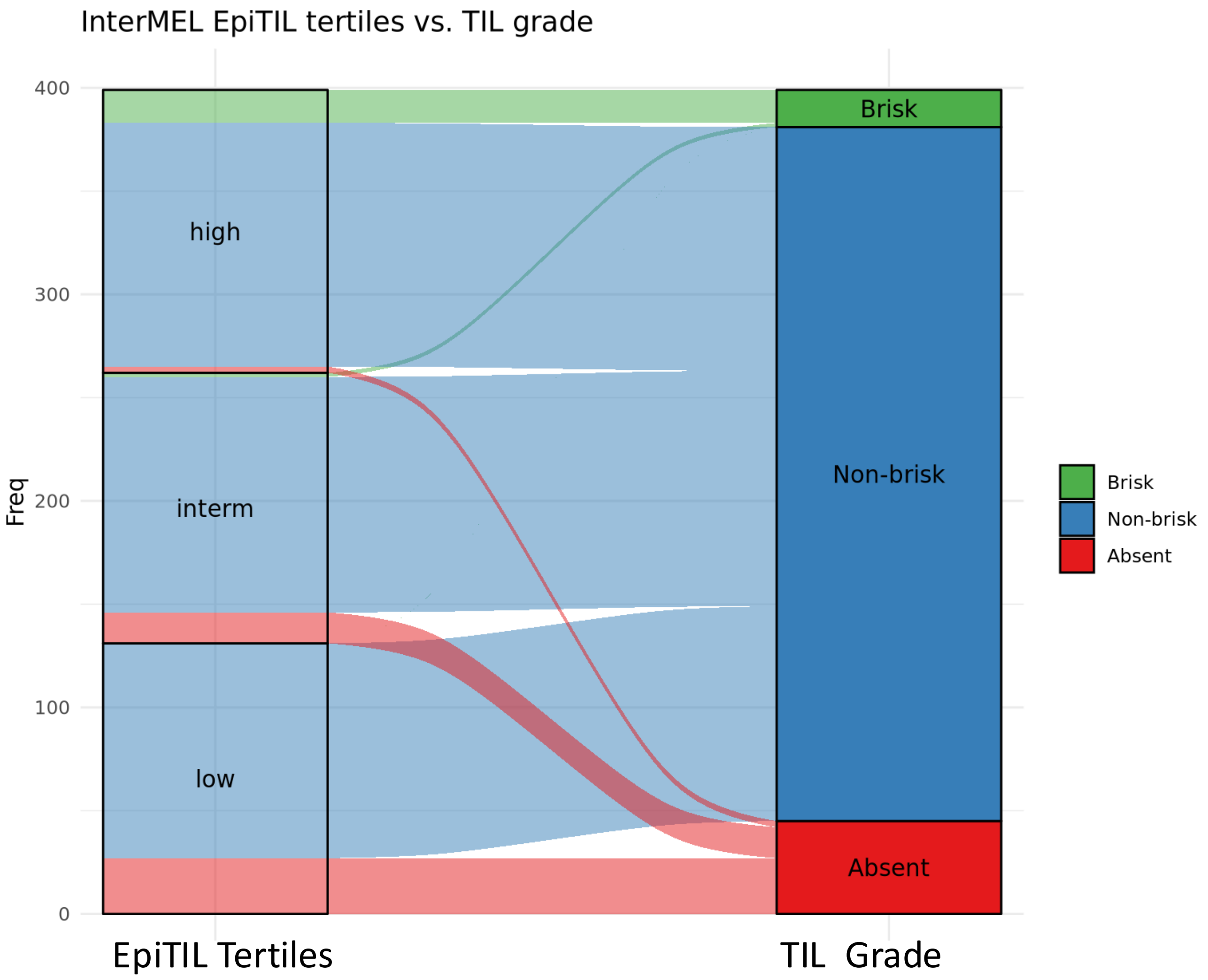

Figure S7: Sankey diagram showing the proportional stratification of TIL grade into EpiTIL tertiles. TIL grade [brisk (green), nonbrisk (blue), absent (red)] are on the right and EpiTIL tertiles (high, intermediate, low) are on the left.

Figure S8

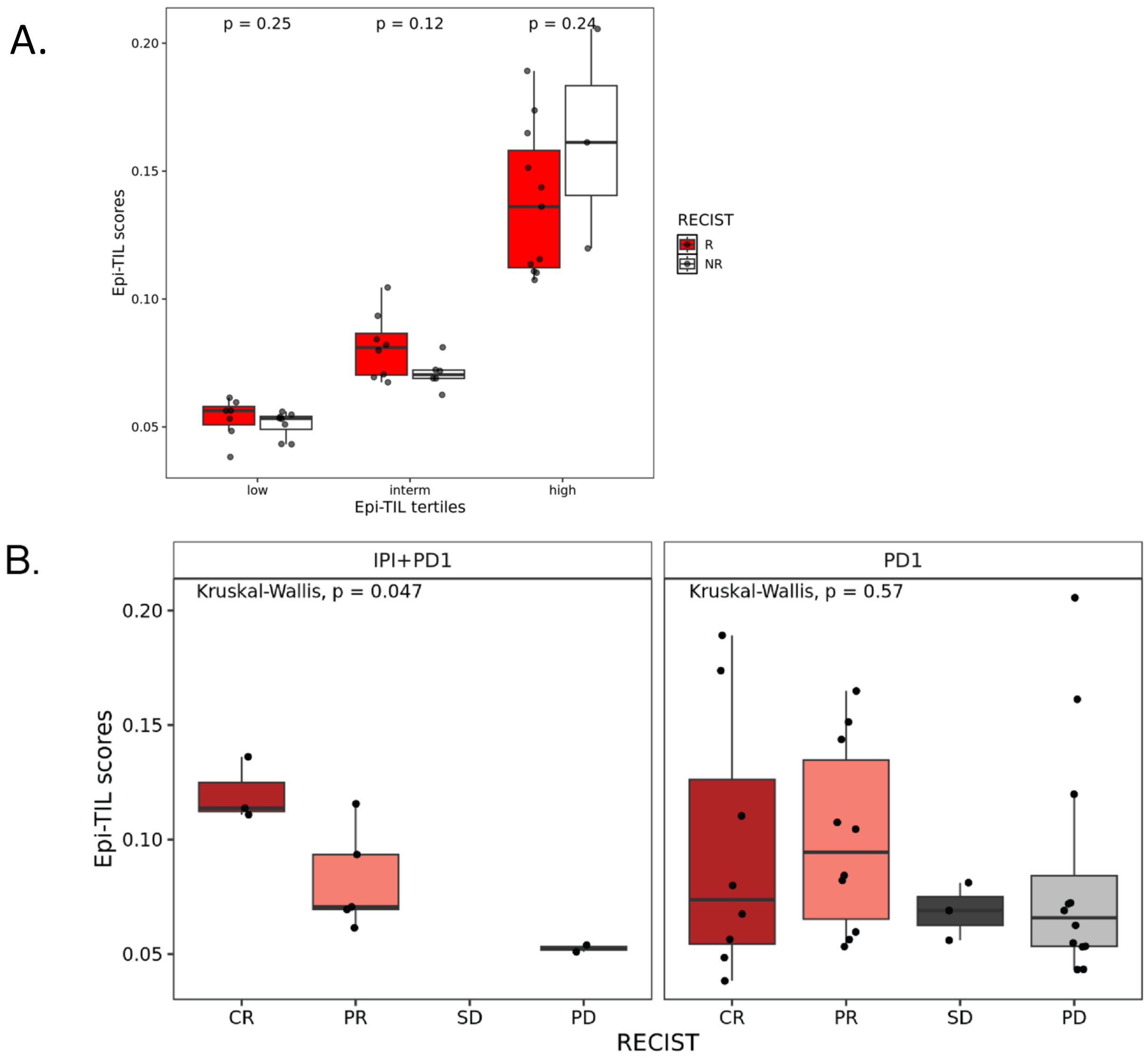

Figure S8: ICI Response in Metastatic Melanoma by EpiTIL scores. (A) Box plots showing ICI responders (R; red) and non-responders (NR; white) plotted by EpiTIL tertiles (high, intermediate, low); p values are listed above each tertile. (B) Box plots showing ICI response by combination therapy (Ipi + PD1) or mono therapy (PD1) for complete response (CR; dark red), partial response (PR; pink), stable disease (SD; dark grey), and progressive disease (PD; light grey) plotted by EpiTIL scores.

Table S1: Characteristics of UNC Melanomas (n=80)

Characteristic	
Sex	Male (61.3%)
Age	>50 yrs (76.3%)
AJCC Stage	Stage I (27.5%), II (40%), III (15%) and IV (17.5%)
Breslow (mm)	1.6 mm (0.37-17mm)
Ulceration	Present (36.6%)
Survival	Died (41.2%), Alive (58.8%)

Table S2: InterMEL Patient Characteristics

Characteristic	N = 422
**Vital Status**	
Died from melanoma <5 years after diagnosis	193 (46%)
Lived >5 years after diagnosis	229 (54%)
**Pathologic Stage**	
II	224 (53%)
III	198(47%)
**TIL Grade**	
Brisk	45 (11%)
Nonbrisk	336 (84%)
Absent	18 (5%)
Missing	23
**MethylClass**	
CIMP	101 (24%)
IM	120 (28%)
LM	201(48%)
Purity	0.44 (0.32, 0.57)

Table S3: InterMEL: Odds rations for 5 years melanoma-specific survival

Table S3. InterMEL Study: Odds ratios for 5 years melanoma-specific survival			
Groups compared	Odds Ratio	95% CI	p value
**TIL grade**			
Absent	ref		
Nonbrisk	2.04	1.09-3.94	0.030
Brisk	8.24	2.31-39.5	0.003
**TIL grade (age + sex)**[1]			
Absent	ref		
Nonbrisk	1.94	1.02-3.76	0.050
Brisk	7.59	2.10-36.7	0.005
**TIL Grade (age + sex + stage)**[2]			
Absent	ref		
Nonbrisk	1.63	0.78-3.44	0.200
Brisk	4.41	1.05-24.2	0.060
**TIL grade (age + sex + stage + EpiTIL)**[3]			
Absent	ref		
Nonbrisk	1.04	0.48-2.78	0.930
Brisk	1.63	0.36-9.35	0.550

Abbreviations: Confidence Interval (CI); Intermediate (IM); Tumor Infiltrating Lymphocytes (TIL)

[1]Base model adjusted for age, sex; [2]Base model + AJCC stage; [3]Base model + AJCC stage + EpiTIL subclass (low, IM, high)

Categorical descriptions: American Joint Committee on Cancer (AJCC); AJCC Category (Cat.) (Gershenwald et al., CA Cancer J Clin. 2017) Cat. 1: IIA +IIIA, Cat. 2: IIB + IIC + IIIB, Cat. 3: IIIC, Cat. 4: IIID; and TIL grade (absent, nonbrisk, brisk) (Clark et al., JNCI, 1981; Busam et al., Am J Clin Pathol 2001).
